# Sickle cell anemia and social justice: a pathway to equitable healthcare in Uganda

**DOI:** 10.1097/MS9.0000000000003910

**Published:** 2025-09-16

**Authors:** Emmanuel Ifeanyi Obeagu

**Affiliations:** Department of Biomedical and Laboratory Science, Africa University, Mutare, Zimbabwe

**Keywords:** genetic disorders, health disparities, sickle cell anemia, social justice, stigma

## Abstract

Sickle cell anemia (SCA) presents a significant yet under-addressed public health burden in Uganda, particularly among marginalized and underserved populations. This review explores how social justice principles can inform equitable healthcare responses to SCA in Uganda. We conducted a structured narrative review using PubMed, Scopus, and African Journals Online as primary databases, with Google Scholar used as a supplementary tool. The review highlights key barriers to care, including limited newborn screening, health infrastructure deficits, sociocultural stigma, and inadequate policy implementation. We critically examined Uganda’s National Sickle Cell Control Program and identified limitations such as low coverage, poor logistics, and fragmented data systems. Drawing on Ghana’s nationally coordinated sickle cell response, we discuss successful community-based models and policy integration as potential frameworks for Uganda. The review advocates for a justice-centered approach that prioritizes health equity, community engagement, and systemic reform. Strengthening Uganda’s SCA response requires not only biomedical interventions but also political commitment and socially responsive health strategies.

## Introduction

Sickle cell anemia (SCA) is a genetic blood disorder that arises due to the inheritance of two sickle cell genes, one from each parent. This condition is characterized by the presence of hemoglobin S, which causes red blood cells to distort into a sickle or crescent shape. These abnormally shaped cells can obstruct blood flow, leading to severe pain, organ damage, and increased susceptibility to infections. SCA is a major public health issue in many parts of the world, particularly in sub-Saharan Africa, where malaria, a selective pressure for the sickle cell trait, is endemic. Uganda is among the countries with the highest burden of SCA, making it a significant focus for healthcare and social justice efforts^[1,2]^. Social justice, as defined in public health, refers to the fair and equitable distribution of resources, opportunities, and privileges within a society, with particular attention to marginalized and vulnerable populations[3]. Uganda’s high prevalence of SCA, affecting approximately 1–2% of the population, translates to around 20 000 new cases each year. This places a considerable strain on the country’s healthcare system, which is already challenged by limited resources and infrastructure. Many individuals with SCA in Uganda do not receive timely or adequate care, leading to high morbidity and mortality rates. The high burden of SCA is further exacerbated by socioeconomic disparities, which limit access to healthcare services, diagnostic facilities, and appropriate treatment[2]. Social justice in healthcare refers to the equitable distribution of resources and opportunities to ensure that all individuals have access to necessary medical care and support, regardless of their socioeconomic status, geographic location, or ethnic background. In Uganda, achieving social justice in the context of SCA involves addressing various social determinants of health that contribute to disparities in care. These determinants include healthcare access, economic barriers, social stigma, and discrimination, all of which negatively impact individuals with SCA and their families[3]. Access to healthcare is a fundamental right, yet many individuals with SCA in Uganda face significant barriers in this regard. Healthcare facilities, particularly in rural and underserved areas, often lack the necessary infrastructure, including diagnostic equipment and trained healthcare professionals, to provide effective care for SCA patients. This results in delayed diagnoses, inadequate treatment, and poor health outcomes. Strengthening healthcare infrastructure is therefore crucial to improving access to care and ensuring that individuals with SCA receive the medical attention they need[4].

**HIGHLIGHTS**

- **Health Equity Advocacy**: Promotes policies ensuring equal access to sickle cell care for all.
- **Reduced Discrimination**: Addresses stigma through education and community sensitization.
- **Inclusive Healthcare**: Integrates sickle cell disease (SCD) services into national health programs.
- **Economic Empowerment**: Supports affected families through financial and social interventions.
- **Patient-Centered Policies**: Encourages participation of SCD patients in healthcare decision-making.

Economic barriers also play a significant role in limiting access to SCA care in Uganda. The cost of medical care, including hospitalizations, medications, and regular check-ups, can be prohibitive for many families, particularly those in low-income brackets. This financial burden often leads to delayed or foregone treatment, exacerbating health disparities and contributing to the high morbidity and mortality rates associated with SCA. Implementing financial support programs and expanding health insurance coverage are essential steps toward alleviating this economic burden and ensuring that all individuals with SCA can access necessary care[5]. Social stigma and discrimination are additional challenges faced by individuals with SCA in Uganda. The visible and debilitating nature of the disease can lead to negative perceptions and attitudes, affecting patients’ mental health, social integration, and employment opportunities. Stigma and discrimination can also discourage individuals from seeking medical care or disclosing their condition, further hindering effective disease management. Increasing public awareness and education about SCA is crucial to reducing stigma, promoting early diagnosis, and encouraging individuals to seek appropriate care[6]. Public awareness and education are key components in the fight against SCA in Uganda. Many people in the country have limited knowledge about the disease, its symptoms, and its genetic nature. This lack of awareness can result in misconceptions, delayed diagnoses, and inadequate treatment. Community outreach, school programs, and media campaigns can play a significant role in educating the public about SCA, promoting early detection, and encouraging individuals to seek medical care[7]. Advocacy and policy development are essential for promoting social justice and improving SCA care in Uganda. Engaging stakeholders, including government agencies, non-governmental organizations (NGOs), healthcare providers, and patient advocacy groups, is crucial for developing and implementing policies that address the unique needs of individuals with SCA. Policies should focus on improving healthcare infrastructure, expanding access to diagnostic and treatment services, and providing financial support for affected families[1]. SCA presents a significant public health challenge in Uganda, compounded by socioeconomic disparities and healthcare inequities. Addressing these issues through a social justice lens requires a multifaceted approach that includes strengthening healthcare infrastructure, enhancing public awareness, providing financial support, investing in research, and advocating for policy development. By bridging gaps in care and support, Uganda can improve the quality of life for individuals with SCA and ensure a more equitable healthcare system for all[8].

## Aim

The aim of this comprehensive strategy for SCA in Uganda is to improve the quality of life for individuals affected by SCA through the implementation of equitable and sustainable healthcare practices, effective public awareness campaigns, and robust policy advocacy.

### Rationale

SCA remains one of the most neglected public health challenges in Uganda, despite its high prevalence and substantial morbidity and mortality. The condition disproportionately affects marginalized communities, particularly in rural and underserved areas, where access to timely diagnosis, comprehensive care, and supportive services is limited. While biomedical research has advanced understanding of the disease, little attention has been given to the social, economic, and systemic factors that perpetuate health disparities among individuals with SCA. Addressing SCA in Uganda requires more than clinical solutions – it demands a social justice approach that confronts structural inequalities, stigma, and resource gaps. Social justice, in the context of healthcare, refers to the fair distribution of resources, equal access to care, and protection of vulnerable populations. This framework is essential for understanding how geographic, economic, cultural, and policy barriers intersect to affect the health outcomes of those living with SCA.

Currently, national efforts – such as the Uganda Sickle Cell Control Program – lack the breadth, funding, and integration necessary to ensure equity in service delivery. Community-based interventions are sparse, newborn screening (NBS) is limited, and public awareness remains low. Without intentional policy reform and systemic change, the cycle of disadvantage will continue. This review aims to bridge the gap between clinical care and health equity by critically examining how social justice principles can be applied to improve SCA outcomes in Uganda. It offers a comprehensive synthesis of the disease burden, identifies the key barriers to equitable care, and explores actionable strategies grounded in justice and inclusivity. In doing so, it contributes to a growing dialogue on the need for socially responsive health systems in low- and middle-income countries.

### Review methods

This narrative review employed a structured literature search to identify peer-reviewed publications, policy reports, and grey literature relevant to SCA and social justice in the Ugandan context. The review aimed to synthesize current knowledge on disease burden, barriers to equitable care, and justice-based interventions.

### Search strategy

A comprehensive search was conducted using three primary academic databases: PubMed, Scopus, and African Journals Online. These databases were selected for their wide coverage of biomedical, social science, and Africa-focused health literature. The search terms included combinations of keywords and MeSH (Medical Subject Headings), such as:

- “sickle cell anemia” OR “sickle cell disease”
- “Uganda”
- “social justice” OR “health equity” OR “health disparities”
- “screening” OR “policy” OR “barriers” OR “healthcare access”

Boolean operators (AND/OR) were applied to refine search results. The initial search was limited to articles published in English between January 2010 and March 2025.

In addition to the core databases, Google Scholar was used as a supplementary tool to capture gray literature, including conference proceedings, governmental reports, and NGO publications not indexed in the primary databases. Reference lists of key articles were also hand-searched to identify additional relevant sources.

### Inclusion and exclusion criteria

Studies were included if they:

- Focused on SCA in Uganda or sub-Saharan Africa with comparable healthcare systems.
- Discussed aspects of health equity, access to care, or social determinants of health.
- Presented primary data, systematic reviews, or policy analyses.

Articles were excluded if they:

- Were not available in full text.
- Focused solely on molecular or genetic mechanisms without reference to healthcare delivery or social context.
- Were published in languages other than English.

The final selection of sources was reviewed for relevance, methodological rigor, and contribution to the overarching themes of social justice, healthcare access, and policy reform in the Ugandan SCA landscape.

### Epidemiology and burden of SCA in Uganda

SCA is a major public health concern in Uganda, with the country experiencing one of the highest prevalence rates globally. The disease affects approximately 1–2% of the Ugandan population, translating to around 20 000 new cases annually. This high prevalence is attributed to the genetic nature of SCA and the historical prevalence of malaria in the region, which has exerted selective pressure for the sickle cell trait (HbAS). The sickle cell trait provides a survival advantage against malaria, but when two carriers reproduce, there is a 25% chance of their offspring inheriting SCA[1]. The distribution of SCA in Uganda is not uniform, with higher prevalence rates observed in the Northern and Eastern regions of the country. These areas have historically had higher malaria transmission rates, which has contributed to the increased frequency of the sickle cell gene. In these regions, the prevalence of the sickle cell trait can be as high as 20%, compared to the national average of 13.3%. This regional variation underscores the need for targeted public health interventions and resources to address the burden of SCA[2]. Despite the high prevalence of SCA, many individuals in Uganda remain undiagnosed or are diagnosed late. NBS for SCA is not universally implemented, leading to delays in diagnosis and initiation of appropriate care. This gap in early diagnosis is a significant challenge, as early intervention can mitigate many of the severe complications associated with the disease. Without timely diagnosis and treatment, children with SCA are at a higher risk of morbidity and mortality, particularly from infections such as pneumonia and malaria, which are common in Uganda[3]. The healthcare infrastructure in Uganda faces substantial challenges in providing adequate care for SCA patients. Many healthcare facilities, especially in rural and underserved areas, lack the necessary diagnostic tools and trained personnel to manage SCA effectively. This results in a heavy reliance on a few specialized centers, predominantly located in urban areas, which can lead to overburdened services and long wait times for patients. The lack of accessible and comprehensive care exacerbates the health disparities faced by individuals with SCA, particularly those from low-income and rural backgrounds[4].

The economic burden of SCA on patients and their families is significant. The costs associated with frequent hospital visits, medications, and managing complications can be prohibitive, especially for low-income families. These economic barriers often lead to delayed or forgone treatment, worsening health outcomes and increasing the risk of severe complications. Additionally, the recurrent nature of SCA crises means that many patients are unable to attend school or maintain employment, further exacerbating the economic impact on families and contributing to cycles of poverty[5]. Social stigma and discrimination compound the challenges faced by individuals with SCA in Uganda. There is a general lack of understanding about the disease, leading to misconceptions and negative attitudes. Patients may be ostracized or face discrimination in various aspects of life, including education, employment, and social interactions. This stigma can also discourage individuals from seeking medical care or disclosing their condition, further hindering effective disease management and support[6]. The psychological impact of SCA on patients and their families is profound. The chronic pain and recurrent hospitalizations associated with the disease can lead to mental health issues such as depression and anxiety. Families often experience significant emotional and financial stress, struggling to cope with the demands of caring for a child with a chronic illness. Support services, including counseling and community support groups, are crucial in helping patients and families manage the psychological burden of SCA[7]. Efforts to address the burden of SCA in Uganda require a comprehensive and multifaceted approach. Strengthening healthcare infrastructure, particularly in rural and underserved areas, is critical to ensuring that all individuals with SCA have access to timely and appropriate care. Public awareness campaigns can help reduce stigma and promote early diagnosis and treatment. Financial support programs and expanded health insurance coverage are necessary to alleviate the economic burden on families and ensure equitable access to care. SCA poses a significant epidemiological and socioeconomic burden in Uganda. Addressing this burden requires a concerted effort to improve healthcare infrastructure, enhance public awareness, provide financial support, and reduce social stigma. By adopting a comprehensive and equitable approach, Uganda can significantly improve the quality of life for individuals with SCA and move toward a more just and effective healthcare system.

## Barriers to social justice in sickle cell disease

### Stigma and societal perceptions

Stigma and societal perceptions surrounding SCA compound the challenges faced by individuals grappling with this genetic disorder in Uganda[8]. Cultural beliefs often shape the way societies perceive and respond to health conditions. The impact of stigma is acutely felt in healthcare settings where individuals with SCA seek medical assistance[9]. Societal attitudes toward individuals with SCA often perpetuate discrimination, limiting opportunities for education, employment, and social inclusion[10]. Efforts to destigmatize SCA require targeted interventions[11]. Community-driven campaigns, educational programs, and advocacy efforts are explored for their potential to shift societal attitudes toward a more compassionate and informed perspective on SCA. Education plays a pivotal role in challenging misconceptions and reducing stigma associated with SCA. The power of personal narratives in humanizing the experiences of individuals with SCA cannot be overstated. Personal narratives serve as a tool to challenge stereotypes, create empathy, and amplify the voices of those affected by SCA.

### Social justice and health disparities

Social justice in healthcare aims to ensure that all individuals, regardless of socioeconomic status, geographic location, or ethnicity, have equitable access to medical services and support. In Uganda, addressing social justice in the context of SCA involves tackling the various social determinants of health that contribute to disparities in care. These determinants include healthcare access, economic barriers, social stigma, and discrimination, all of which significantly impact individuals with SCA and their families. One of the primary social justice issues in Uganda’s healthcare system is the disparity in access to medical services. Rural and underserved areas often lack the necessary infrastructure, such as clinics, laboratories, and trained healthcare professionals, to effectively diagnose and treat SCA. This results in delayed diagnoses, inadequate treatment, and poor health outcomes for patients. Strengthening healthcare infrastructure is essential to improving access to care. This involves building more healthcare facilities, equipping them with the necessary diagnostic tools, and training healthcare workers to manage SCA. In urban areas, while healthcare facilities are more available, they are often overwhelmed by the high demand for services, leading to long wait times and inadequate care. Urban healthcare centers require additional support and resources to manage the high volume of patients and provide timely and effective treatment for SCA. Ensuring that both rural and urban areas have adequate healthcare resources is crucial for achieving equitable healthcare access^[8–10]^.

Economic barriers significantly hinder access to SCA care in Uganda. The cost of medical care, including hospitalizations, medications, and regular check-ups, can be prohibitive for many families, particularly those in low-income brackets. This financial burden often leads to delayed or forgone treatment, exacerbating health disparities and contributing to the high morbidity and mortality rates associated with SCA. Implementing financial support programs and expanding health insurance coverage are essential steps toward alleviating this economic burden and ensuring that all individuals with SCA can access necessary care. Furthermore, the economic impact of SCA extends beyond medical costs. The recurrent nature of SCA crises means that many patients are unable to attend school or maintain employment, further exacerbating the economic impact on families and contributing to cycles of poverty. Support programs that provide financial assistance for education and employment opportunities for individuals with SCA and their families can help mitigate these economic challenges. Social stigma and discrimination are additional challenges faced by individuals with SCA in Uganda. There is a general lack of understanding about the disease, leading to misconceptions and negative attitudes. Patients may be ostracized or face discrimination in various aspects of life, including education, employment, and social interactions. This stigma can also discourage individuals from seeking medical care or disclosing their condition, further hindering effective disease management and support. Addressing social stigma requires comprehensive public awareness and education campaigns to inform the public about SCA, its causes, symptoms, and treatment. These campaigns should aim to dispel myths and misconceptions about the disease and promote a more inclusive and supportive environment for individuals with SCA. Community outreach, school programs, and media campaigns can play a significant role in educating the public and reducing stigma^[11,12]^.

The psychological impact of SCA on patients and their families is profound. The chronic pain and recurrent hospitalizations associated with the disease can lead to mental health issues such as depression and anxiety. Families often experience significant emotional and financial stress, struggling to cope with the demands of caring for a child with a chronic illness. Support services, including counseling and community support groups, are crucial in helping patients and families manage the psychological burden of SCA. Providing comprehensive psychological and social support services is essential for improving the quality of life for individuals with SCA and their families. These services should be integrated into the healthcare system and made accessible to all patients, regardless of their geographic location or economic status. Advocacy and policy development are essential for promoting social justice and improving SCA care in Uganda. Engaging stakeholders, including government agencies, NGOs, healthcare providers, and patient advocacy groups, is crucial for developing and implementing policies that address the unique needs of individuals with SCA. Policies should focus on improving healthcare infrastructure, expanding access to diagnostic and treatment services, and providing financial support for affected families^[13–16]^.

### Economic impact

The economic impact of SCA in Uganda is profound, affecting not only individuals and their families but also the broader healthcare system and national economy. The costs associated with managing SCA are multifaceted, encompassing direct medical expenses, indirect costs related to loss of productivity, and broader socioeconomic implications. Addressing these economic challenges requires comprehensive strategies to alleviate the financial burden on affected families and to optimize the allocation of resources within the healthcare system. The direct medical costs of SCA are significant and include expenses related to hospitalizations, medications, regular check-ups, and emergency care. Frequent hospital visits for pain management, blood transfusions, and treatment of complications such as infections or organ damage contribute to high out-of-pocket expenses for families. In Uganda, where healthcare resources are limited and health insurance coverage is not widespread, these costs can be prohibitive for many families, leading to delayed or forgone treatment and worsening health outcomes. To alleviate these costs, the government and healthcare providers can work towards reducing the price of essential medications through bulk purchasing agreements and partnerships with pharmaceutical companies. Expanding health insurance schemes to cover SCA-related treatments and services can also reduce out-of-pocket expenses and improve access to necessary care^[8–10]^.

Beyond direct medical expenses, SCA imposes substantial indirect costs on individuals and their families. The recurrent nature of SCA crises often results in significant time lost from work and school, leading to reduced productivity and income. Adults with SCA may struggle to maintain stable employment due to frequent illness and hospitalizations, while parents of children with SCA may need to take time off work to care for their sick children. This loss of productivity can have a cascading effect on family finances, contributing to cycles of poverty and economic instability. Providing social support programs, such as paid family medical leave and educational accommodations for children with SCA, can help mitigate these indirect costs and support affected families in maintaining economic stability. The economic burden of SCA extends beyond individual households to the broader healthcare system and national economy. High rates of hospitalization and the need for specialized care place a significant strain on the healthcare system, diverting resources that could be used for other public health initiatives. The economic impact of SCA also affects national productivity and economic growth, as a substantial portion of the workforce may be unable to contribute fully due to the debilitating effects of the disease. Investing in preventive measures, such as NBS programs and early intervention strategies, can reduce the long-term economic burden of SCA by decreasing the incidence of severe complications and improving health outcomes. Additionally, integrating SCA care into primary healthcare services can optimize resource allocation and reduce the need for expensive specialized care^[11,12]^.

To address the economic impact of SCA, implementing comprehensive financial support programs and expanding health insurance coverage are essential. Subsidizing the cost of medications, hospital visits, and other related expenses can help families access the care they need without financial strain. Health insurance schemes should be designed to cover a broad range of SCA-related treatments and services, ensuring that all individuals with SCA can receive timely and effective care. Public–private partnerships and international collaborations can play a critical role in funding and supporting these financial support programs. Leveraging resources from various stakeholders can enhance the sustainability and reach of these initiatives, ensuring that they can effectively address the economic challenges faced by individuals with SCA and their families. Community-based economic support programs can provide additional assistance to families affected by SCA. Microfinance initiatives, vocational training programs, and small business grants can empower families to improve their economic stability and reduce their reliance on external financial support. These programs can also promote economic resilience by helping families develop diverse income sources and build financial literacy. Engaging community organizations and local businesses in these initiatives can enhance their effectiveness and sustainability. By fostering a supportive community environment, these programs can help families affected by SCA overcome economic challenges and achieve greater financial independence^[13,14]^.

### Bridging gaps: strategies for improved care and social justice

Addressing the challenges of SCA in Uganda requires a multifaceted approach that tackles both the healthcare needs and the socioeconomic factors contributing to health disparities. Implementing strategies to bridge these gaps will enhance the quality of life for individuals with SCA and promote social justice in healthcare. Enhancing the capacity of healthcare facilities, particularly in rural and underserved areas, is crucial for improving access to SCA care. This includes building and equipping clinics with diagnostic tools such as hemoglobin electrophoresis machines and training healthcare workers to diagnose and manage SCA effectively. Additionally, establishing more specialized centers for SCA care in both urban and rural areas can help distribute the patient load and provide comprehensive care closer to patients’ homes. Implementing universal NBS programs for SCA is essential for early diagnosis and intervention. Early detection allows for timely medical care and management, reducing the risk of severe complications and improving long-term health outcomes. Government and healthcare providers should collaborate to ensure that screening programs are accessible and integrated into routine neonatal care across the country. Ensuring that essential medications, such as hydroxyurea and pain management drugs, are readily available and affordable is critical for managing SCA. The government can negotiate with pharmaceutical companies to reduce drug prices and include SCA medications in the national essential medicines list. Additionally, establishing a national blood donation program can help ensure a steady supply of safe blood for transfusions, which are often necessary for SCA patients^[17,18]^.

Alleviating the economic burden on families affected by SCA requires implementing financial support programs and expanding health insurance coverage. Subsidizing the cost of medications, hospital visits, and other related expenses can help families access the care they need without financial strain. Expanding national health insurance schemes to cover SCA-related treatments and services can also reduce out-of-pocket expenses and promote equitable access to healthcare. Increasing public awareness about SCA is essential for reducing stigma and promoting early diagnosis and treatment. Educational campaigns through community outreach, school programs, and media can inform the public about the genetic nature of SCA, its symptoms, and the importance of seeking medical care. Educating communities about the disease can help dispel myths, reduce discrimination, and encourage supportive attitudes toward individuals with SCA. Comprehensive psychological and social support services are vital for individuals with SCA and their families. Counseling services, support groups, and community-based programs can help patients and families cope with the emotional and psychological challenges associated with the disease. Integrating these services into the healthcare system and making them accessible to all patients can improve mental health outcomes and overall quality of life. Investing in research is critical for understanding the epidemiology, treatment outcomes, and social impact of SCA in Uganda. Establishing national registries and conducting epidemiological studies can provide valuable data to inform public health strategies and policies. Research efforts should focus on identifying effective treatment protocols, understanding the genetic basis of the disease, and evaluating the impact of social determinants on health outcomes. Effective advocacy is essential for driving policy development and implementation to address the needs of individuals with SCA. Engaging stakeholders, including government agencies, NGOs, healthcare providers, and patient advocacy groups, can help create a supportive environment for policy change. Policies should focus on improving healthcare infrastructure, expanding access to diagnostic and treatment services, and providing financial support for affected families. Community involvement is crucial for the successful implementation of SCA programs and interventions. Empowering communities to take an active role in addressing SCA can enhance the effectiveness of public health initiatives. Community health workers (CHWs) and volunteers can play a significant role in raising awareness, providing education, and supporting patients and families. A comprehensive national strategy for SCA should be developed to coordinate efforts across different sectors and ensure a holistic approach to disease management. This strategy should include components such as healthcare infrastructure development, public awareness campaigns, financial support mechanisms, research and data collection, and policy advocacy. Collaboration between the government, healthcare providers, NGOs, and international partners is essential for the successful implementation of a national strategy^[19–21]^.

### Community engagement and advocacy

Community engagement and advocacy are critical components in the fight against SCA in Uganda. Effective community engagement ensures that initiatives are culturally appropriate, widely accepted, and sustainable, while advocacy efforts can drive policy changes and increase resource allocation. Together, these strategies can enhance public awareness, reduce stigma, improve healthcare access, and ultimately lead to better health outcomes for individuals with SCA. One of the primary goals of community engagement is to raise public awareness about SCA. Misconceptions and a lack of understanding about the disease contribute to stigma and discrimination against those affected. Public awareness campaigns can play a pivotal role in educating communities about the genetic nature of SCA, its symptoms, and the importance of early diagnosis and treatment. Community outreach programs can include educational workshops, seminars, and health fairs conducted in local languages and tailored to the cultural context. Leveraging local media, including radio, television, and social media platforms, can also help disseminate accurate information about SCA widely. Collaborating with community leaders, religious institutions, and local organizations can enhance the credibility and reach of these awareness campaigns. Empowering individuals with SCA and their families through education and support is crucial for effective disease management. Patient support groups can provide a platform for sharing experiences, coping strategies, and emotional support. These groups can also serve as a conduit for disseminating information about available healthcare services, financial assistance programs, and new treatment options. Training CHWs to provide basic SCA education and support can further empower patients and families. CHWs can conduct home visits, offer guidance on managing SCA symptoms, and help patients navigate the healthcare system. This grassroots approach ensures that support reaches even the most remote and underserved areas^[22–24]^.

Advocacy efforts are essential for driving policy changes that improve SCA care and support. Engaging with policymakers, government officials, and other stakeholders can help raise the profile of SCA as a public health priority. Advocacy groups can lobby for the implementation of national NBS programs, the inclusion of SCA treatments in national health insurance schemes, and increased funding for SCA research and healthcare infrastructure. Forming coalitions with other health advocacy groups can amplify the voice of the SCA community and increase the impact of advocacy efforts. These coalitions can work together to organize public demonstrations, policy dialogues, and press conferences to highlight the needs of individuals with SCA and the importance of equitable healthcare access. Building strong partnerships with community organizations, local businesses, and NGOs can enhance the effectiveness of community engagement and advocacy efforts. These partnerships can facilitate resource sharing, collaborative program development, and the pooling of expertise. For example, partnering with local schools can integrate SCA education into the curriculum, ensuring that young people learn about the disease from an early age. Collaborations with local businesses can lead to the creation of employment opportunities and vocational training programs for individuals with SCA and their families, helping to mitigate the economic impact of the disease^[25–27]^.

Community engagement is vital for promoting research and data collection efforts. Encouraging community participation in research studies can help gather accurate epidemiological data, understand treatment outcomes, and identify social determinants of health affecting individuals with SCA. These data are essential for informed decision-making and effective policy development. Engaging communities in the research process, through community advisory boards or participatory research methods, ensures that studies are conducted ethically and that findings are relevant to the local context. This approach can also build trust between researchers and communities, increasing the likelihood of successful study implementation and knowledge dissemination. Ensuring the sustainability of community engagement and advocacy initiatives requires ongoing capacity building. Training local leaders, health workers, and community members to take ownership of SCA programs can foster long-term commitment and resilience. Providing continuous education and resources to these local champions ensures that they remain effective advocates and educators. Capacity building efforts should also focus on developing the skills of SCA patients and their families, enabling them to become self-advocates and active participants in their healthcare. This empowerment can lead to more effective disease management and a stronger, more vocal SCA community. Monitoring and evaluation (M&E) are critical for assessing the effectiveness of community engagement and advocacy efforts. Establishing clear indicators and metrics for success allows stakeholders to track progress, identify challenges, and make data-driven adjustments to programs. Regular M&E can also demonstrate the impact of these initiatives to funders and policymakers, securing ongoing support and resources. Incorporating feedback mechanisms, such as community forums and surveys, ensures that the voices of individuals with SCA and their families are heard and considered in program evaluation. This participatory approach can enhance the relevance and effectiveness of engagement and advocacy strategies^[28–30]^.

U.S. Food and Drug Administration approved two milestone treatments, Casgevy and Lyfgenia, representing the first cell-based gene therapies for the treatment of sickle cell disease in patients 12 years and older. Additionally, one of these therapies, Casgevy, is the first FDA-approved treatment to utilize a type of novel genome editing technology, signaling an innovative advancement in the field of gene therapy[31]. This will need some awareness in health institutions and communities for the effective utilization of these therapies.

## The role of men in SCA

SCA is a genetic blood disorder that profoundly affects individuals and families worldwide. While the focus of much of the discourse around SCA often centers on the medical aspects of the disease, the role of men in the management and impact of SCA is equally significant. Men are integral to the fight against SCA, both as patients and as key players in the support systems for those affected. This narrative explores the multifaceted roles that men play in the context of SCA, examining their responsibilities, challenges, and contributions to both care and advocacy. In the realm of SCA, fathers assume crucial roles as caregivers and providers for their families. Their involvement can be seen in various aspects of managing the disease, from accompanying their children to medical appointments to providing emotional and financial support. For many fathers, the diagnosis of SCA in a child marks the beginning of a profound journey, one that requires a balance of strength, empathy, and resilience. Men also play a significant role in advocacy efforts aimed at increasing awareness and improving conditions for those affected by SCA. Advocacy is a crucial component of social justice, and men’s participation in this area can drive significant change in public perception and policy[32].

Men also contribute to the understanding and treatment of SCA through their professional roles as researchers, doctors, and healthcare workers. Their work in the field directly impacts the quality of care available to SCA patients and the development of new treatments and strategies for disease management. Men’s roles extend into the realm of healthcare decisions, where they often serve as partners in the decision-making processes regarding treatment plans and health strategies. This partnership is crucial for ensuring that all family members are involved in managing the disease effectively. Beyond the practical aspects of care and advocacy, men also play an essential role as emotional support providers for their families and communities. This emotional labor is often unspoken but is a crucial element of managing the psychological impact of SCA. In addition to their roles as caregivers, advocates, and professionals, men also contribute to SCA management through educational efforts. They engage in training programs that aim to enhance knowledge about the disease and improve care practices. Men’s roles extend into the policy arena, where they can influence health policies and advocate for systemic changes that benefit SCA patients. Their efforts in policy advocacy are crucial for securing resources and shaping healthcare priorities. While men play diverse and significant roles in the context of SCA, they also face unique challenges. These challenges include balancing personal and professional responsibilities, overcoming stigma, and navigating the complexities of healthcare systems[32].

## Special funding for SCA

SCA represents a significant public health challenge, particularly in countries like Uganda, where the disease burden is high. While traditional funding mechanisms for healthcare often focus on broad, general needs, special funding initiatives for SCA are crucial for addressing the specific needs of individuals affected by this genetic disorder. Historically, funding for SCA has been uneven, with a greater focus on diseases perceived as more prevalent or more urgent. However, over the past few decades, there has been a growing recognition of the importance of dedicated funding for SCA. Special funding initiatives have emerged to bridge gaps in research, treatment, and education, reflecting a shift toward more targeted health interventions. In the early 2000s, SCA was often overshadowed by more visible diseases like HIV/AIDS or malaria. However, organizations like the World Health Organization and the National Institutes of Health (NIH) began to prioritize SCA through special funding programs. One landmark initiative was the launch of the Sickle Cell Disease Treatment Demonstration Program by the NIH in 2007, which aimed to improve care for SCA patients through focused research and funding. This program was instrumental in setting a precedent for dedicated SCA funding and has since paved the way for other special funding efforts. Governments and international organizations have recognized the need for special funding to address SCA’s unique challenges. These initiatives provide essential financial support for a range of activities, from basic research to patient care programs. In Uganda, the Ministry of Health has implemented special funding programs aimed at improving SCA care. For example, the “National Sickle Cell Program” established in 2010 provides funding for NBS, treatment services, and public education. This program is supported by the Ugandan government in partnership with international donors such as the United Nations Children’s Fund. The funding supports the establishment of screening facilities, training for healthcare professionals, and the development of public awareness campaigns. This initiative exemplifies how government and international partnerships can pool resources to address the specific needs of SCA patients. The Global Fund to Fight AIDS, Tuberculosis and Malaria, although primarily focused on these diseases, has also supported SCA initiatives through its “Diseases of Poverty” program. This program offers grants for the development of healthcare infrastructure and disease management strategies in low-income countries. For instance, a grant from the Global Fund helped support the establishment of a comprehensive SCA treatment center in Kampala, which provides diagnostic services, treatment, and support for SCA patients. Private sector companies and philanthropic organizations play a significant role in funding special projects for SCA. These contributions often focus on innovative research, patient support services, and awareness campaigns^[21,33–36]^.

The Sickle Cell Disease Association of America (SCDAA) is a prominent example of a philanthropic organization that has made substantial contributions to SCA funding. Through donations, fundraising events, and grants, SCDAA supports research initiatives, patient support programs, and educational outreach efforts. One notable project funded by SCDAA is the “Sickle Cell Disease Research Grant Program,” which provides grants to researchers exploring new treatment options and disease management strategies for SCA. This program has funded numerous studies that have advanced our understanding of SCA and led to the development of new therapies. Pharmaceutical companies such as Novartis and Pfizer have also been involved in special funding for SCA. Novartis, for instance, has supported the “Sickle Cell Disease Initiative,” which funds research on new drug treatments and supports clinical trials for innovative therapies. Additionally, Pfizer’s “Global Health Innovation Fund” has provided grants for projects aimed at improving SCA care in under-resourced regions. These private sector efforts are critical for advancing treatment options and expanding access to new therapies for SCA patients. Community-based fundraising efforts are vital for supplementing official funding sources and supporting local SCA initiatives. These grassroots efforts often involve events and campaigns that raise awareness and generate financial support for SCA-related causes^[37–40]^.

In Uganda, community-based organizations like the “Sickle Cell Foundation Uganda” have spearheaded fundraising efforts through events such as charity runs, community fairs, and benefit dinners. These events not only raise funds but also engage the community in SCA awareness and advocacy. For example, the “Sickle Cell Awareness Walk” organized by the Foundation has become an annual event that raises both funds and awareness for SCA. The success of such events illustrates how community-driven efforts can complement larger funding initiatives and foster a sense of collective responsibility toward managing SCA. Special funding has had a profound impact on the care and management of SCA. This funding supports a range of activities that improve patient outcomes, advance research, and drive advocacy efforts. The introduction of hydroxyurea as a treatment for SCA is a direct result of special funding for research and clinical trials. Grants from organizations like the National Heart, Lung, and Blood Institute facilitated research that demonstrated the efficacy of hydroxyurea in reducing sickle cell crises and improving patient quality of life. The availability of hydroxyurea as a treatment option has been a game-changer for SCA management, and this advancement was made possible through targeted funding efforts. The “Sickle Cell Disease Management Program” funded by the Bill & Melinda Gates Foundation is another example of impactful special funding. This program focuses on improving SCA care in sub-Saharan Africa by providing resources for disease management, patient education, and healthcare infrastructure development. The program’s success in increasing the availability of SCA treatments and training healthcare professionals demonstrates how special funding can lead to tangible improvements in patient care. While special funding initiatives have made significant contributions to SCA care, there are challenges and opportunities associated with these funding mechanisms. One of the primary challenges is ensuring that funds are allocated effectively and reach the communities in need. There can be issues with the distribution of resources, with funds sometimes failing to reach local healthcare facilities or being used inefficiently. Additionally, there is often competition for funding among various health priorities, which can limit the resources available for SCA. Despite these challenges, there are opportunities for expanding special funding efforts. Increasing awareness among donors about the specific needs of SCA patients can lead to more targeted and effective funding initiatives. Additionally, fostering collaborations between governments, international organizations, and private sector partners can enhance the effectiveness of funding programs and ensure that resources are used to their fullest potential^[41–45]^.

## Comparative perspectives: lessons from Ghana’s national sickle cell strategy

While Uganda has taken steps toward addressing SCA, comparative analysis with other sub-Saharan African nations reveals valuable insights. Notably, Ghana has emerged as a regional leader in implementing structured national SCA programs that prioritize early detection, public education, and integrated care.

- **National NBS program:** Ghana introduced a nationwide NBS initiative for SCA as early as 1995, starting with pilot programs in Kumasi and Accra. With continued support from the Ministry of Health and international collaborators, this effort has expanded to include numerous regional hospitals and health centers. Unlike Uganda’s pilot programs with limited geographical reach, Ghana’s screening strategy is embedded within the national immunization framework, thereby ensuring more consistent coverage and follow-up care[29].
- **Decentralized and community-based services:** Ghana’s model emphasizes decentralization of care, bringing diagnostic and treatment services closer to affected populations. CHWs are trained to raise awareness, identify at-risk families, and provide psychosocial support. This grassroots involvement enhances community trust, reduces stigma, and encourages health-seeking behavior – challenges that Uganda continues to grapple with.
- **Public education and advocacy:** A key strength of Ghana’s approach lies in its robust public education campaigns. Government-supported media initiatives and school-based outreach programs aim to increase awareness of sickle cell inheritance, reduce stigma, and promote voluntary screening among couples. In contrast, Uganda’s public health messaging around SCA remains limited, fragmented, and often confined to clinical contexts[29].
- **Integration with national policy:** Ghana formally integrated SCA control into its national health policy framework, ensuring dedicated funding and long-term sustainability. This policy alignment has facilitated multi-sectoral collaboration and consistent political will. Uganda, while acknowledging SCA as a priority, has yet to fully mainstream it within broader national health and social protection agendas.
- **Outcomes and impact:** Evidence from Ghana’s program shows improved early diagnosis rates, increased survival among affected children, and growing public acceptance of screening. These outcomes underscore the effectiveness of a rights-based, community-inclusive approach to SCA management.
- **Implications for Uganda:** Uganda can draw key lessons from Ghana’s experience by expanding NBS, investing in community health structures, launching sustained public education campaigns, and integrating SCA services into national health policies. Strengthening inter-country collaboration within the East and West African contexts may also promote shared learning and resource optimization for better outcomes.

## Limitations of current national programs

Despite notable efforts to address SCA in Uganda – particularly through the Ministry of Health’s National Sickle Cell Control Program – significant limitations continue to undermine the effectiveness and equity of these interventions. These challenges span structural, operational, and sociocultural domains, impeding comprehensive care delivery and population-wide prevention efforts.

- **Low NBS coverage:** Although Uganda launched a pilot NBS program for SCA in selected districts, national coverage remains critically low. According to the Ministry of Health’s Sickle Cell Surveillance Report (2021), less than 20% of newborns are screened annually, primarily in urban and peri-urban centers. This limited outreach leaves many rural and high-burden communities undiagnosed, delaying early interventions that could significantly reduce childhood morbidity and mortality^[46,47]^.
- **Logistical and infrastructure constraints:** Healthcare facilities in remote districts often lack the laboratory capacity, trained personnel, and consistent supply chains necessary for diagnosis and follow-up. Many regional hospitals are unable to conduct confirmatory testing or provide hydroxyurea therapy, while district-level health centers are generally unprepared to manage chronic complications. Transportation costs, poor road networks, and lack of integration between screening sites and treatment facilities further compound these challenges[46].
- **Inadequate data systems and surveillance:** Program monitoring is hindered by weak data collection systems and fragmented reporting mechanisms. Without real-time, reliable epidemiological data, the national program struggles to allocate resources effectively or monitor progress. There is also limited tracking of patient outcomes, making it difficult to evaluate the long-term impact of existing interventions[47].
- **Sociocultural and stigmatic barriers:** Social stigma and misinformation about SCA remain widespread in many communities. Misconceptions regarding inheritance patterns, spiritual beliefs surrounding chronic illness, and discrimination against individuals with SCA often discourage families from seeking screening or treatment. Fear of social exclusion may also lead to underreporting or denial of a child’s diagnosis. These cultural barriers are seldom addressed by programmatic outreach, which tends to focus on biomedical messaging[48].
- **Policy gaps and funding limitations:** Although Uganda has recognized SCA as a public health priority, implementation has been constrained by limited political commitment and inadequate budgetary allocation. Donor support has largely driven pilot interventions, with minimal sustainable investment from national sources. Furthermore, SCA is often overshadowed by higher-profile diseases such as HIV/AIDS and malaria, resulting in inconsistent policy focus[49].

## Recommendations on SCA and social justice: bridging gaps in Uganda

### Strengthen public awareness and education campaigns

Launch comprehensive public education campaigns to raise awareness about SCA, focusing on the genetic nature of the disease, its symptoms, and the importance of early diagnosis. Many individuals in Uganda may lack understanding of SCA and its implications. Educating the public can reduce stigma, encourage early testing, and promote informed decision-making regarding carrier status and reproductive choices. Develop and disseminate educational materials through community health centers, schools, and media. Organize community outreach programs with local leaders, educators, and health professionals. Incorporate sickle cell education into school curriculums and community health workshops.

### Enhance access to sickle cell testing and diagnosis

Expand access to affordable and accessible sickle cell testing and diagnostic services, particularly in rural and underserved areas. Early diagnosis is crucial for managing SCA effectively. Many individuals, especially in remote areas, do not have access to testing services. Increase the availability of sickle cell screening programs in primary healthcare settings. Provide training for healthcare workers on SCA diagnosis and management. Collaborate with NGOs and international health agencies to support screening initiatives.

### Improve treatment and care services

Invest in the development and enhancement of healthcare infrastructure to provide consistent and comprehensive care for individuals with SCA. Effective management of SCA requires regular medical care, access to medications, and emergency treatment, which are often lacking in many regions. Increase funding for SCA treatment programs and medication supplies. Establish specialized sickle cell clinics and support the training of healthcare professionals in sickle cell care. Develop a national treatment protocol for SCA and ensure adherence to best practices.

### Support research and innovation

Encourage and fund research focused on new treatments, management strategies, and the development of affordable therapies for SCA. Ongoing research is essential for discovering new treatment options and improving the quality of life for individuals with SCA. Promote partnerships between local universities, research institutions, and international research bodies. Provide grants and funding opportunities for research focused on SCA. Facilitate knowledge exchange and collaboration among researchers and clinicians.

### Address social and economic inequalities

Develop social safety nets and economic support programs for families affected by SCA. Families with SCA face significant economic burdens due to medical costs and lost income. Addressing these inequalities is a matter of social justice. Create financial assistance programs for medical expenses and provide support for families with sick children. Establish educational scholarships and vocational training for individuals affected by SCA. Advocate for policies that provide financial relief and support for caregivers of individuals with SCA.

### Promote policy advocacy and reform

Advocate for the creation and implementation of national policies and laws that support individuals with SCA and promote social justice. Strong governmental support and legal frameworks are necessary for addressing the challenges of SCA effectively. Engage with policymakers to advocate for the inclusion of SCA in national health priorities. Support the development of legislation that ensures access to treatment, education, and support for individuals with SCA. Monitor and evaluate the implementation of policies to ensure they meet the needs of affected communities.

### Foster community engagement and empowerment

Involve community leaders, patient advocacy groups, and affected individuals in the planning and implementation of SCA programs and initiatives. Community involvement ensures that programs are culturally relevant, effective, and responsive to the needs of those affected. Establish forums for dialogue between affected individuals, community leaders, and health professionals. Support patient advocacy groups and empower them to take an active role in SCA initiatives. Encourage grassroots efforts to address local challenges and promote collective action.

## Conclusion

Community engagement and advocacy are integral components of a comprehensive approach to addressing the challenges faced by individuals with SCA in Uganda. By fostering inclusive support networks, promoting grassroots initiatives, recognizing the contributions of NGOs and CHWs, advocating for policy changes, and leveraging digital platforms, this review aims to inspire collaborative efforts that bring about positive change for the SCA community in Uganda. It is our hope that the insights presented will encourage sustained engagement, advocacy, and a collective commitment to creating a more supportive and understanding environment for those affected by SCA.

SCA in Uganda presents a complex interplay of medical, social, and economic challenges that demand a comprehensive and compassionate response. This review has delved into critical aspects of the SCA landscape, including healthcare accessibility, stigma, economic impact, and the role of community engagement and advocacy. As we conclude, several key reflections and recommendations emerge: The journey toward addressing SCA in Uganda requires an unwavering commitment to social justice, inclusivity, and equity. By integrating cultural competence into healthcare, dismantling societal stigmas, fostering economic empowerment, championing grassroots initiatives, and promoting collaboration, we can create a more supportive environment for individuals with SCA. This paper serves as a call to action, urging all stakeholders to join hands in shaping a future where the challenges of SCA are met with understanding, empathy, and a collective commitment to the well-being of all.

## Data Availability

Not applicable as this a narrative review.

## Research registration unique identifying number (UIN)

Not applicable as this is a narrative review.
